# Participation of older newly-diagnosed cancer patients in an observational prospective pilot study: an example of recruitment and retention

**DOI:** 10.1186/1471-2407-9-277

**Published:** 2009-08-10

**Authors:** Martine TE Puts, Johanne Monette, Veronique Girre, Christina Wolfson, Michele Monette, Gerald Batist, Howard Bergman

**Affiliations:** 1 Solidage Research Group, Université de Montréal/McGill University Research Group on Frailty and Aging, Montreal, Canada; 2 Department of Epidemiology, Biostatistics and Occupational Health, McGill University, Montreal, Canada; 3 Division of Geriatric Medicine, Sir Mortimer B. Davis-Jewish General Hospital, McGill University, Montreal, Canada; 4 Institut Curie, Department of Medical Oncology, Paris, France; 5 Division of Clinical Epidemiology McGill University Health Centre (MUHC), Montreal, Canada; 6 Segal Cancer Centre, Jewish General Hospital, McGill University, Montreal, Canada; 7 Department of Health Administration, Université de Montréal, Montreal, Canada

## Abstract

**Background:**

There have been few prospective observational studies which recruited older newly-diagnosed cancer patients, and of these only some have reported information on the number needed to screen to recruit their study sample, and the number and reasons for refusal and drop-out. This paper reports on strategies to recruit older newly-diagnosed cancer patients prior to treatment into an observational prospective pilot study and to retain them during a six-month period.

**Methods:**

Medical charts of all patients in the Segal Cancer Centre aged 65 and over were screened and evaluated for inclusion. Several strategies to facilitate recruitment and retention were implemented. Reasons for exclusion, refusal and loss to follow-up were recorded. Descriptive statistics were used to report the reasons for refusal and loss to follow-up. A non-response analysis using chi-square tests and t-tests was conducted to compare respondents to those who refused to participate and to compare those who completed the study to those who were lost to follow-up. A feedback form with open-ended questions was administered following the last interview to obtain patient's opinions on the length of the interviews and conduct of this pilot study.

**Results:**

3060 medical charts were screened and 156 eligible patients were identified. Of these 112 patients participated for a response rate of 72%. Reasons for refusal were: feeling too anxious (40%), not interested (25%), no time (12.5%), too sick (5%) or too healthy (5%) or other reasons (5%). Ninety-one patients participated in the six-month follow-up (retention 81.3%), seven patients refused follow-up (6.2%) and fourteen patients died (12.5%) during the course of the study. The median time to conduct the baseline interview was 45 minutes and 57% of baseline interviews were conducted at home. Most patients enjoyed participation and only five felt that the interviews were too long.

**Conclusion:**

It was feasible to recruit newly-diagnosed cancer patients prior to treatment although it required considerable time and effort. Once patients were included, the retention rate was high despite the fact that most were undergoing active cancer treatment.

## Background

Cancer is a common health problem for older persons. Nevertheless, the manifestations and course of cancer in this older population have not been well investigated and remain poorly understood [1-6]. Moreover, with the aging of the population both the absolute and the relative number of older patients affected by cancer can be expected to increase in the future [7,8]. There is increasing interest in conducting research on health and functional status in older cancer patients [9,10]. It has been suggested that frailty might be a useful concept to identify vulnerable older patients *at risk *of adverse outcomes of treatment who then might benefit from comprehensive geriatric assessment [11,12]. Frailty represents a state of reduced homeostasis and resistance to stress, due in part to aging, which leads, in turn, to increased vulnerability and risk of adverse outcomes although there is no consensus on the definition. Fried et al [13] have developed a now commonly used approach to measure frailty. The five characteristics of their frailty phenotype are: weakness, poor endurance, reduced physical activity, slow walking speed, and unintentional weight loss during the preceding year. Cognitive impairment and depressed mood have recently been added [14,15]. Unlike their non-frail contemporaries, frail older individuals appear unable to withstand stressors, such as environmental stress, injury, and acute illness. These stressors may provoke a downward spiral, whereby the frail older individual is unable to recover and return to the baseline state [13,14,16]. In older patients, cancer treatments, especially chemotherapy, are considered strong stressors that will reveal which patients have sufficient functional reserve to regain stable homeostasis [3,17]. To our knowledge, there has been no prospective observational study investigating the usefulness of the concept of frailty in the field of geriatric oncology.

There have been many studies that have reported that older patients are underrepresented in cancer clinical trials due to a variety of reasons [6,18-27]. Furthermore, investigators have reported that recruiting older patients to any clinical trial is difficult due to co morbidity, cognitive problems, sensory deficits, transportation issues, study burden and influence of significant others [28-35]. As observational studies do not bring personal advantage or direct benefits for patients as do clinical trials, the reasons for participation and refusal might be different than for clinical trials. Newly-diagnosed cancer patients may also be asked to participate in a cancer clinical trial at the same time as being asked to participate in an observational study which may increase the burden on the older patients and may affect response rates and retention rates in both studies. Few observational studies have reported information on the number needed to screen to recruit the study sample, or the number and reasons for refusal and drop-out [36,37]. Furthermore, most of the studies have been cross-sectional with small sample sizes and/or with only one type of cancer diagnosis included [38-42]. The studies with older cancer patients have defined their older patients with no cut-off based on age [36,37], 65 years and over [39,41] or 70 years and over [38,40,42]. More information on recruitment and retention strategies may facilitate or increase participation of older newly-diagnosed cancer patients in studies on health and functional status.

In the context of an observational prospective pilot-study conducted to describe the health and vulnerability of older newly-diagnosed cancer patients and the adverse outcomes of treatment, we evaluated and report our recruitment and retention strategies to optimize their use for future larger studies. As we were interested whether the concept of frailty is useful to identify older patients *at risk *of adverse outcomes of cancer treatment, it was crucial to conduct the baseline interview *prior to *cancer treatment. The window of opportunity for recruitment of older newly-diagnosed cancer patients was thus narrow, making recruitment more challenging. The aim of this study was to examine the feasibility of recruitment older newly-diagnosed cancer patients prior to treatment into an observational prospective pilot study and to retain them during a six-month follow-up period.

## Methods

### Study population

**Figure 1:**
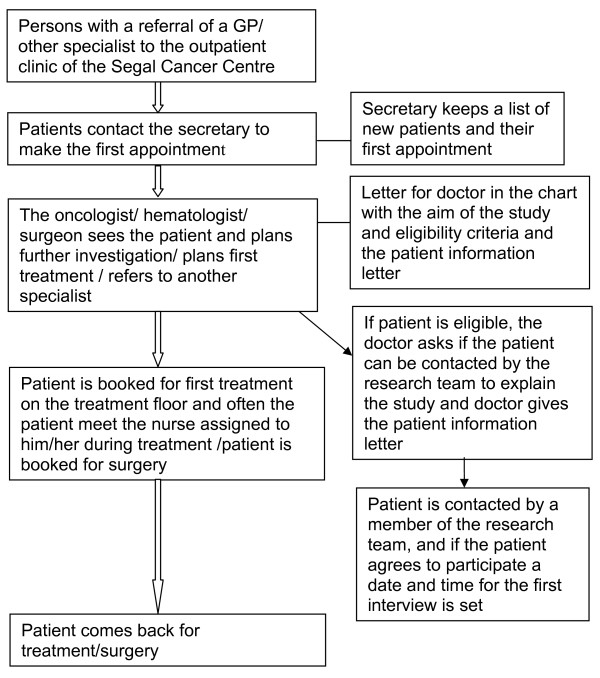
**Flow chart of patients in the Segal Cancer Centre and the recruitment**.

The study sample was recruited at the Segal Cancer Centre which is a comprehensive cancer centre. It is a referral center for local and regional patients. It serves the adult population referred by other doctors and other hospitals in Quebec for further investigation and cancer treatment [see Figure 1]. Each year more than 5,000 patients are seen for a total of more than 70,000 visits per year. The Segal Cancer Centre includes surgeons, medical oncologists, haematologists, radiation oncologists and pulmonary oncologists involved in patient's cancer treatments. The inclusion criteria for this study were: patients referred to the Segal Cancer Centre of the Jewish General Hospital, McGill University, Montreal, Canada, aged 65 and older, with a new diagnosis of solid tumour with or without metastasis (i.e. breast, colorectal, or lung cancer) or haematological malignancy (i.e. lymphoma and myeloma), who had not received cancer treatment in the previous five years. The only exception was for those who had started hormonal therapy less than 1 month prior to the study. These particular cancer diagnoses were chosen as they are among the most common cancers diagnosed in older Canadians [43]. The age cut-off was chosen since this cut-off is often used in longitudinal aging studies and has been used previously in studies with older cancer patients [39,41]. Exclusion criteria were: unable to speak English or French, estimated life expectancy less than 3 months, unable to give informed consent because of cognitive impairment. The study was approved by the Research Ethics Committee of the Jewish General Hospital, McGill University, Montreal, Canada. All participants gave written informed consent.

### Study description

The baseline interview took place *prior to *cancer treatment and the participants were followed for six months with face-to-face follow-up interviews at three and six months and telephone interviews at 1.5 and 4.5 months [see Additional file 1]. The baseline interview consisted of questions on sociodemographics, health (lifestyle factors) and functional status, comorbid conditions, use of medications, disability in instrumental and daily living, quality of life, anxiety and depression, and use of health care [44-51]. In addition, to evaluate the frailty markers, nutrition (including weight and height), grip strength and gait speed were assessed and cognitive functioning tests were administrated [52-56]. The third and fifth face-to-face follow-up interviews included the same questions with the exception of the sociodemographic data which was only included in the baseline interview. The second and fourth interview which was conducted by telephone included health and functional status, disability in daily and instrumental activities of daily living, quality of life and the use of health care.

### Recruitment strategies

Patients were identified through the lists of appointments at the oncology, pulmonary oncology and haematology oncology clinics. A letter to the physician including the inclusion and exclusion criteria and a patient information letter were added to the chart of each eligible patient (see Table 1 with recruitment strategies by diagnosis). If the patient was found to be eligible, the treating physician then asked the patient if he/she could be contacted by telephone or be seen after the consultation by a member of the research team to explain the study and discuss potential participation. A member of the research team invited the patient to participate and when they agreed, an interview date was set with the patient choosing to do the interview at home or at the hospital. The Department of colorectal surgery is located outside the Segal Cancer Centre and for logistical reasons it was not possible to identify potential eligible patients through the lists of appointments. The surgeons of patients newly-diagnosed with colorectal cancer identified eligible patients and asked them if they could be contacted by the research team. These patients were seen by a member of the research team at the inpatient ward the day they were admitted for their surgery planned for the following day. If they agreed to participate the interview was done the day of admission. Screening of the medical charts, contacting the participants and the interviews were done by MP (trained as a nurse), VG (medical oncologist) and MM (trained as an occupational therapist). MP and VG started recruitment and interviews and MM assisted with the conduct of interviews. Recruitment began on March 1^st ^2007 and ended on January 31^st ^2008 for all cancers except colorectal cancer for which recruitment ended on May 1^st ^2008.

**Table 1:** Overview of Recruitment Strategies by Cancer Diagnosis

Strategy used	Lung cancer	Breast cancer	Lymphoma/Multiple myeloma	Colorectal cancer
Screening charts for clinics in Segal Cancer Centre	X	X	X	X
Attending tumor board	X	X	X	X
Letter for MD with inclusion and exclusion criteria in chart and patient info letter in chart of potential patient	X	X	X	X (in Segal Cancer Centre, not in the Department of colorectal surgery)
After consultation contact MD to verify eligibility and to ask the MD if the patient agreed to be contacted	X	X	X	X
Contact the secretary of each of the departments each week to see list of new patients		X	X	X
Contact the surgeons to verify new eligible patient				X
Contact the nurse/MD to confirm eligibility and approached the patient the day before the surgery				X

### Retention strategies

A combination of retention strategies was used. Participants were offered home visits or transportation to the hospital and they were interviewed at their preferred time. Participants were offered transportation/compensation of costs for a hospital visit if they had no other appointment that day. Furthermore, they were offered the opportunity to complete the face-to-face interviews in two parts if it was not possible for them, for whatever, reason to complete it in one interview.

As far as possible participants were interviewed by the same person and all participants were sent holiday cards while on study. Patients were also give fridge magnets with the telephone numbers how to reach the study team. Prior to each telephone interview the patient was sent a letter reminding them of the date and time of the interview. Also the questionnaire was sent with this letter to facilitate the interview. For participants with visual or hearing impairment the patients were asked what they would prefer: a face-to-face interview or to receive the questionnaire with a pre-stamped return envelope to complete themselves instead of the telephone interview.

Lastly, during the informed consent procedure, participants were asked permission to contact a proxy in case the research team could not contact them during the follow-up period or they would not feel well enough to do the interview.

### Data collection

The medical charts of all patients in the Segal Cancer Centre aged 65 and over were screened and evaluated for inclusion. The reasons for exclusion were recorded for potentially eligible patients (i.e. a new diagnosis of cancer). Furthermore, the reasons for refusing to participate by eligible patients, the reasons for drop-out, the number of meetings and time required to complete the interview and reasons for postponement were recorded. The time needed to complete the interview was defined as the time between the start and the end time of the interview. If the interview was completed in two parts the total time was summed. The time needed to complete the interviews was only calculated for those respondents who completed the entire interview, i.e. those who completed *all *of the different measurements and questionnaires and who had a time available. Interviews that were not entirely completed are referred to as incomplete interviews throughout the article. Postponement was recorded when the respondent did not want to schedule the interview or do the planned interview when he or she was contacted. In these cases the reason for postponement was asked. The number of days necessary to complete the entire study was calculated for the participants who participated in all five interviews.

To compare the baseline characteristics of those who completed the study, died while on study or refused further follow-up we used the baseline sociodemographic information collected (age, sex, marital status, living situation (grouped as alone or not alone), and born in Canada (yes or no)). Information on diagnosis, stage of disease, treatment plan proposed and whether or not they were enrolled in a clinical trial was obtained from the medical records. The extent of disease at diagnosis was classified as early (stage 0–2) or advanced (stage 3–4). The Eastern Oncology Cooperative Group Performance Scale (ECOG PS) (grouped as 0, 1 and ≥ 2) [49] was used to classify functional status. Anxiety and depression were assessed using the Hospital Anxiety and Depression Scale (HADS; a score >10 as possible impairment) [50], cognition using the Mini Mental State Examination [52] and the Montreal Cognitive Assessment Tool (MoCa) (a score of 26 or below on either MMSE or MoCa indicating cognitive impairment) [53], Instrumental activities of daily living (IADL disability, used as no IADL disability or ≥ 1 IADL disability) was measured using the Older American Resources and Services [46]. The Katz index was used for disability in activities of daily living (ADL disability, used as no ADL disability vs. ≥ 1 ADL disability) [47] and the functional comorbidity index was used the calculate the number of comorbid conditions [44].

#### Feedback from patients

Patients who completed the follow-up of 6 months were asked to provide feedback by filling out a one-page questionnaire after the last interview. Participants were asked to gauge the strength of their agreement with the statement "I found the length of the interview too long" (strongly disagree to strongly agree) for both the telephone and face-to-face follow-up interview. Furthermore, they were asked three questions: 1) Are there questions that you found irrelevant, unpleasant or too difficult? If yes, could you explain why? 2) Are there topics that we did not discuss in this study, but you find important for your health and functional status (meaning your ability to do your normal daily activities)? 3) Other comments or suggestions with regard to the conduct of this study?

### Statistical analysis

Descriptive statistics including means and proportions were used to describe the characteristics of those who were newly-diagnosed but excluded because they did not fulfill all inclusion criteria, of the patients who refused and of the patients who participated in the study. A non-response analysis was conducted using chi-square tests and two-sample t-tests. Descriptive statistics were used to describe the mean duration and the reasons for postponement of interviews and the opinion of patients on the length of the interviews. To examine if the feasibility (defined as number of days needed to complete the study, duration of the interviews, number of postponed interviews and number of interviews completed in one or two meetings or incomplete) of the study was different based on the characteristics of the patients, the participants were grouped in several ways. The participants were divided into groups based on median age at baseline (74.1 years), sex, living situation, born in Canada, diagnosis, extent of disease, number of comorbid conditions, IADL disability, and ADL disability. Descriptive techniques such as frequencies and crosstabs were used to compare if the groups based on the characteristics mentioned above differed with regard to the feasibility of the study. The answers to the open-ended feedback questions were transcribed, grouped and counted. Statistical analyses were performed using SPSS version 15.0 (SPSS Inc., Chicago, Illinois, USA).

## Results

**Figure 2:**
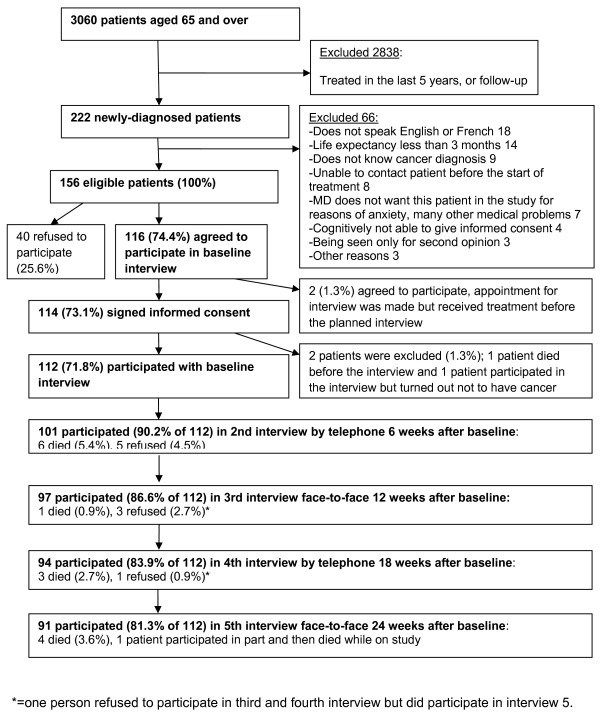
**Overview Recruitment and retention in the study**.

From March 1^st ^2007 until May 1^st^, 2008, 3060 medical charts of patients aged 65 and over were screened in the Segal Cancer Centre; see Figure 2. Of those 3060 patients, 2838 were excluded (ineligible) because they were treated for cancer in the past 5 years, they were being seen for follow-up appointments after cancer treatment or the patient was investigated for cancer but did not have cancer. There were 222 newly-diagnosed patients to review for further eligibility. Of those 222, 66 were excluded (see Figure 2 for the reasons) leaving 156 eligible patients. Of these, 116 patients agreed to participate. 114 signed informed consent and two persons had to be excluded because they received treatment before the planned interview took place. Of the 114 patients who signed informed consent, one patient died before the baseline interview. A second patient completed the baseline interview but following a second opinion the cancer diagnosis was changed to a non-malignant diagnosis and the patient was excluded. Finally, 112 participants completed the baseline interview (72% of all eligible patients).

Of the 40 patients (25.6%) who refused to participate, 16 reported that they felt overwhelmed/sad by the diagnosis, were too anxious in relation to the start of their cancer treatment, 5 patients did not feel well enough to participate, 5 patients had no time (due to care giving responsibilities, moving or work, or start of treatment next day), 2 patients felt too healthy, 10 persons were not interested, 1 patient refused due to study burden as the patient was also enrolled in a clinical trial and the last person refused due to denial of the cancer diagnosis. However, half of them indicated that they would be willing to participate after the start of treatment (i.e. knowing then what the treatment would be like).

The baseline characteristics of those who participated, those who refused to participate and those who were excluded are presented in Table 2. The only statistically significant difference between those who participated and those who refused was that participants were less often married/living common-law (p = 0.006).

**Table 2:** Characteristics of Participants, those who Refused or were Excluded

Characteristic	Participated N = 112 (%)	Refused to participate N = 40 (%)	Excluded N = 70 (%)
**Mean age at screening (SD)**	73.7 year (6.0)	74.4 year (5.8)	76.2 year (6.6)
**Sex (%)**			
Female	78 (69.6)	21 (52.5)	39 (55.7)
Male	34 (30.4)	19 (47.5)	31 (44.3)
**Language of the patient (%)**			
French	41 (36.6)	8 (20.0)	12 (17.1)
English	71 (63.4)	32 (80.0)	22 (31.4)
Neither	0	0	21 (30.0)
No information	0	0	15 (21.4)
**Marital status (%)**			
Married/living common-law	62 (55.4)	31 (77.5)	45 (64.3)
Widowed	25 (22.3)	7 (17.5)	12 (17.1)
Separated/divorced	19 (17.0)	0	2 (2.9)
Single	6 (5.4)	0	6 (8.6)
No Information	0	2 (5.0)	5 (7.1)
**Type of cancer (%)**			
Lung	27 (24.1)	17 (42.5)	28 (40.0)
Breast	44 (39.3)	11 (27.5)	21 (30.0)
Colorectal	20 (17.9)	7 (17.5)	11 (15.7)
Lymphoma & myeloma	21 (18.8)	5 (12.5)	10 (14.3)
**Treatment proposed (%)**			
Watchful waiting	2 (1.8)	1 (2.5)	11 (15.7)
Surgery	54 (48.2)	19 (47.5)	28 (40.0)
Chemotherapy	42 (37.5)	15 (37.5)	14 (20.0)
Radiation therapy	10 (8.9)	4 (10.0)	9 (12.9)
Hormonal therapy	4 (3.6)	1 (2.5)	4 (5.7)
Other	0	0	4 (5.7)
**Cancer diagnosis (%)**			
First diagnosis	100 (89.3)	34 (85.0)	62 (88.6)
Recurrence	2 (1.8)	4 (10.0)	6 (8.6)
New diagnosis, not first cancer diagnosis	10 (8.9)	2 (5.0)	2 (2.9)

The baseline characteristics of participants who completed the study, died during the study or refused further follow-up interviews are presented in Table 3. Due to the small number of patients who refused, statistical analyses were not conducted comparing these groups. During the study, 14 participants died (12.5%) before the last interview and one patient died following completion of the first half of the interview. As shown in Table 3, those who died seemed to be in worse health and functional status at the baseline interview compared to those who completed the study or refused further follow-up.

**Table 3:** Overview of those who Completed, Refused or Died during the Study

Characteristic	Completed the study N = 90 (%)	Died during study N = 15 (%)	Refused follow-up N = 7 (%)
**Mean age at baseline interview (SD)**	73.6 year SD (6.2)	72.9 year SD (4.6)	76.7 year SD (6.0)
**Sex**			
Female	66	9	6
Male	24	6	1
**Marital status**			
Married or living common-law	46	12	4
Widowed	22	2	1
Separated/divorced	18	0	1
Single	4	1	1
No Information	0	0	0
**Lives alone**			
Yes	37	5	3
No	53	10	4
**Born in Canada**			
Yes	50	11	4
No	40	4	3
**Type of cancer/alive at end of study and completed study**			
Lung	19/19 (100%)	8	0
Breast	41/44 (93.2%)	0	3
Colorectal	14/18 (77.8%)	24	4
Lymphoma	13/13 (100%)	1	0
Multiple myeloma	3/3 (100%)	0	0
**Extent of disease at diagnosis**			
Early (stage 0–2)	55	2	6
Advanced (stage 3–4)	35	13	1
**Enrolled into clinical trial**			
Yes	7	14	7
No	83	1	0
**Treatment proposed**			
Watchful waiting	2	0	0
Surgery	47	1	6
Chemotherapy	29	13	0
	9	1	0
Radiation therapy Hormonal therapy	3	0	1
**Cancer diagnosis**			
First diagnosis	79	14	7
Recurrence	2	0	0
New diagnosis, not first diag.	9	1	0
**Ecog Performance scale at baseline**			
0	65 (72.2)	2 (13.3)	5 (71.4)
1	14 (15.6)	6 (40.0)	2 (28.6)
2	9 (10.0)	3 (20.0)	0
3	2 (2.2)	4 (26.7)	0
4.	0	0	0
Mood impairment (HADS A or D ≥ 10)	18 (20.0)	7 (46.7)	1 (14.3)
Cognitive impairment (MMSE ≤ 26 or Moca ≤ 26)	3 (3.3)	3 (20.0)	2 (28.6)
IADL disability (OARS)	27 (30.0)	8 (53.3)	4 (57.1)
ADL disability (Katz)	7 (7.8)	4 (26.7)	1 (14.3)
Mean number of self-reported comorbid conditions (Functional comorbidity Index)	2.0 (SD 1.9)	3.3 (SD 2.5)	1.9 (SD 1.2)

Seven participants (6.3%) refused follow-up after the baseline interview. There was an additional patient who refused the third and fourth interview due to a lack of time but participated in the final interview. The reasons for refusal were not longer interested (3), no time (1), too overwhelmed by diagnosis and treatment (2), sick husband (1).

Sixty four (57.1%) of all baseline interviews were conducted at home and the remainder were conducted in the hospital, see Table 4. During the course of the study, 61 interviews were postponed by 44 participants. The median time needed to complete the baseline interview (n = 87) was 45 minutes (Interquartile range (IQR) 40–55), 10 minutes for the second interview (N = 77) (IQR 8–15), 45 minutes for the third interview (N = 70) (IQR 35–50), 10 minutes for the fourth interview (N = 70) (IQR 10–15) and 40 minutes for the fifth interview (N = 71) (IQR 35–55). The results of the feasibility of the study according to patients' characteristics showed only some minor differences were observed with not one group for whom participation was less feasible [see Additional file 2].

**Table 4:** Location of Interview and Number of Interviews Needed to Complete the Questionnaire

Interview 1 Baseline Face-to-face N = 112	Number of interviews
Face-to face interview at home	64 (57.1%)
Face-to-face interview at the hospital	48 (42.9%)
Complete interview and interview time available	87 (77.7%)
**Interview 2 Telephone interview after 6 weeks N = 101**	
Interview by telephone	65 (64.4%)
In hospital face-to-face interview	14 (13.9%)
Returned self-administrated questionnaire by mail	9 (8.9%)
Returned self-administrated questionnaire in hospital	13 (12.9%)
**Number of Interviews 2 postponed and reason**	**16 (100%)**
Not feeling well	5 (31.3%)
Postponed to combine with next hospital visit	2 (12.5%)
Away for holiday	1 (6.3%)
Admitted to hospital	3 (18.8%)
Had not received questionnaire by mail	3 (18.8%)
For other reasons (too busy)	2 (12.5%)
**Interview 3 Face-to-face interview after 12 weeks N = 97**	
Face-to face interview at home	45 (46.4%)
Face-to-face interview at the hospital	45 (46.4%)
Filled out questionnaire at home and measurements in hospital	6 (6.2%)
Returned self-administrated questionnaire by mail*	1 (1.0%)
Complete interview and interview time available	78 (80.4%)
**Number of Interviews 3 postponed and reason**	**14 (100%)**
Not feeling well	4 (28.6%)
Postponed to combine with next hospital visit	3 (21.4%)
Away for holiday	3 (21.4%)
Admitted to hospital	1 (7.1%)
For other reasons (too busy)	3 (21.4%)
**Interview 4 telephone interview after 18 weeks N = 94**	
Interview by telephone	56 (59.6%)
In hospital face-to-face interview	14 (14.9%)
Returned questionnaire by mail*	8 (8.5%)
Returned questionnaire in clinic	16 (17.0%)
**Number of Interviews 4 postponed and reason**	**10 (100%)**
Not feeling well	4 (40.0%)
Illness/death family or friends	1 (10.0%)
Postponed to combine with next hospital visit	1 (10.0%)
Admitted to hospital	2 (20.0%)
Had not received questionnaire by mail	2 (20.0)
**Interview 5 Face-to-face interview after 24 weeks N = 91**	
Face-to face interview at home	46 (50.5%)
Face-to-face interview at the hospital	35 (36.1%)
Face-to-face interview in Rehab center	1 (1.1%)
Returned self-administrated questionnaire by mail*	1 (1.1%)
Filled out questionnaire at home, measurements in clinic	8 (8.8%)
Complete interview and interview time available	71 (78.0%)
**Number of Interviews postponed and reason**	**21 (23.1%)**
Not feeling well	4 (19.0%)
Illness/death family or friends	3 (14.3%)
Postponed to combine with next hospital visit	5 (23.8%)
Away for holiday	4 (19.0)
Admitted to hospital	3 (14.3%)
For other reasons (too busy)	2 (9.5%)

* One respondent moved to Australia and continued completing the questionnaires.

During the study, 2 participants became very ill and proxy interviews were conducted (both participants had lung cancer). For two other participants with cognitive impairment a proxy respondent completed the telephone interview while the face-to-face interviews were conducted with the patient and the proxy together. Only two participants used the transportation offered to come to the hospital for an interview. Nobody used the offer to be compensated for transportation costs to the hospital.

In addition, patients were asked their opinion on participating in this study and 84 participants provided feedback (see Table 5). Most did not identify specific questions that they felt were unpleasant or too difficult but did mention that nothing was unpleasant or too difficult. The majority of patients indicated that in their opinion there was no item or topic missing. A third of patients indicated that they had enjoyed participating in this study.

**Table 5:** Feedback obtained from participants

Agreement with the statement "Interviews were too long"	Face-to-face interviews N = 84 (%)	Telephone interviews N = 84 (%)
Strongly disagree	37 (44.0%)	34 (40.5%)
Disagree	41 (48.8%)	45 (53.6%)
No opinion	1 (1.2%)	0
Agree	1 (1.2%)	2 (2.4%)
Strongly agree	4 (4.8%)	3 (3.6%)
**Which questions were too difficult or unpleasant?**		**Number of patients who reported this**
Cognitive tests were too easy or irrelevant		6
Gait speed test, felt shy walking in clinic where other people can see the person walk		1
The interview was too easy		2
Most of questions are not relevant as patient did not feel sick, more suited for more sick patients		2
All questions were relevant		3
The questions were okay (not too strenuous)		3
Too much questions		2
The wide variety of questions makes it interesting		1
**Which items/topics with regard to health and functional status are missing?**		**Number of patients who reported this**
Living situation (are you happy where you live?)		1
Perceptions of treatment		1
		**Number of patients who reported this**
Social support/care received from family and friends		2
Loneliness		1
**Which items/topics with regard to health and functional status are missing?**		**Number of patients who reported this**
Physical activity/hobbies (changes in type and amount of activities)		2
Nutritional problems including change in taste		1
Future (life after treatment)		2
**Other comments or suggestions with regard to the conduct of this study**		**Number of patients who reported this**
I enjoyed participating in this study		30
Comments with regard to specific hospital services that are lacking		2

## Discussion and conclusion

Our results show that it was feasible to recruit newly diagnosed cancer patients prior to treatment although this did require considerable time and effort. Although no information was recorded systematically on the number of hours necessary to recruit potential participants by members of the research team, we estimate that on average it took 3 hours to recruit the potential eligible subjects. This estimate does not include the time needed to assess eligibility. One member of the research team worked fulltime on the recruitment and conduct of interviews. We found it necessary to modify our strategies depending on cancer type. It was more difficult to identify and recruit eligible patients with colorectal cancer compared to the other cancer diagnoses for which the research team had access to the clinic lists. For colorectal cancer the treating surgeon had to alert us when they had a new patient. Regardless of strategies implemented to remind physicians, we found that it was important to be present in the clinics when physicians see their patients to reinforce the reminder. We found that at the time of recruitment it was important to meet the patient in the physician's office so that the patient would know who was going to call them to explain the study. This finding is in keeping with the work of others [28]. We also observed that patients wanted to know the treatment plan *before *they could decide whether or not to participate in this pilot study. During the informed consent procedure a number of questions were asked about the possibility of withdrawing from the study. It was stressed many times that they could withdraw at any time without providing a reason. Despite these initial concerns, during the study this was not a major issue as only two participants dropped out of the study because of the burden of treatment. We also noticed that some patients and family members had different views about whether or not they should participate in the study. In some cases, family members were concerned about the study burden in combination with the cancer treatment even when the patients wanted to participate. When this arose, this concern was successfully addressed by also giving family members all the study information to help them understand the study. Interestingly, in a few cases, patients refused to participate and subsequently their family members asked if they could participate as a proxy, a strategy not included in the study design.

It was important to actively recruit patients after they had agreed to be contacted for the study as none of the patients contacted the research team for information about the study. This is in agreement with Townsley et al. [27] who conducted a survey among elderly patients concerning their willingness to participate in cancer clinical trials and found that most elderly patients were willing to consider participation but did not appear to actively seek information about trials. Our overall recruitment was 72% of eligible patients in spite of the limited time window available to recruit patients. This rate is about the same as reported in other studies with older cancer patients. It is higher than another study with newly-diagnosed cancer patients with breast, colon, lung or colorectal cancer who were recruited within 6 weeks of initial surgery or within 2 weeks of initiating chemotherapy or radiation therapy who reported a response rate of 51% [36-38]. However, a high number of patients had to be screened to recruit those patients. A review on attrition in large longitudinal aging studies (>1000 participants) around the world showed initial response rates of 63 to 93% [57]. Our response rate compares very favourably considering that this is a population of newly-diagnosed cancer patients recruited before the start of treatment.

We compared the participants on several characteristics to identify potential differences in the feasibility of recruitment and retention in this pilot study. There were only minor differences observed. It seemed that those with a poorer functional status needed more time to complete the interviews, the study overall and required more meetings to complete an interview. Similar findings were reported by Pijls-Johannesma et al[58] in a study comparing different quality of life instruments. They found that patients with lung cancer who all had a poor performance status, needed assistance more often to complete the assessment tool than patients with breast cancer. In addition, we found that patients born outside Canada, required more effort to complete an interview and more often had an incomplete interview. Patients were included in the study if they could understand and speak English or French and the study material was provided in English and French. However, some of the foreign-born participants found the questionnaires were too difficult and this may have led to the higher number of incomplete interviews. The cognition tests and the HADS were most often missing for foreign-born patients. In the 2006 census http://www.statcan.ca, 19.5% of Canada's population was born outside Canada. For future studies it is important to pay attention to the feasibility of study design and measurement tools of foreign-born older adults.

We noted that once enrolled in the study, the retention rate was high (81%) despite the fact that the majority of participants were undergoing active cancer treatment. Most patients did not feel that the interviews were too long. From the feedback received from patients, most had enjoyed participating in the study. In the few prospective studies published with older cancer patients, the attrition rate ranged from 2% to 37%, in part due to study differences in baseline age and disease status, treatment regimes and length of follow-up time (ranging between 6 months to 1 year follow-up) [38,40,41,59-61]. The review on attrition in large longitudinal aging studies [57] showed that attrition was associated with increasing age and those who are in worse health. In our study, attrition due to refusal was highest among patients with colorectal cancer which might be due to the fact circumstances and different method of recruitment and attrition due to death was highest amongst patients with lung cancer. These findings are in agreement with the study of Neumark et al. who found that dropout was highest among patients with colorectal and lung cancer [37]. At a University teaching hospital newly-diagnosed cancer patients may also be asked to participate in a cancer clinical trial at the same time as being asked to participate in an observational study which may increase the burden on the older patients and may affect response rates and retention rates in both studies. Unfortunately we did not have the information on how often the patients who refused to participate were asked to participate in a clinical trial. However, one patient who refused to participate told us that she was participating in a clinical trial and thought the burden of both studies would be too much. In addition, one physician asked that we not contact an eligible patient because he wanted to enrol the patient in a clinical trial and thought both studies would be too much. Nevertheless, none of our dropouts during the study were participating in a clinical trial. Future studies should take into account the double study burden of participating in both an observational study and clinical trial.

The planning of interviews with participants undergoing cancer treatment needed to be flexible as a significant number of participants had to postpone and reschedule interview appointments due to a variety of reasons. This is in agreement with the study of Shipman et al [62] where patients with advanced lung or colorectal cancer (n = 20) were interviewed to determine their views about participating in serial questionnaires studies. These authors also reported that the interview appointments were often postponed and patients reported that an interview duration of 30–45 minutes in length would be preferred with a preference for face-to-face interviews which is in agreement with our findings [62]. It was also important for participants, as far as possible, to have the interviews done by the same person and to offer home visits and transportation. For each face-to-face data collection about half of the interviews were conducted at home. Furthermore, participants in our study who were born outside Canada and who were not native English or French speakers preferred to have a family member present during the interviews in case they might not understand the question. In most of these cases, interviews were done in the oncology centre while participants were with a family member waiting to see their oncologist and this is partly the reason why some interviews were postponed until the next appointment.

To our knowledge this is one of the first studies with older newly-diagnosed cancer patients examining the recruitment and retention strategies for an observational study that begins prior to cancer treatment. A limitation of our study is that we only recruited participants at one cancer centre and the recruitment and retention process in other hospitals may differ. Due to the small numbers of patients who refused, statistical analyses to compare the characteristics of the refusers to the participants were not possible. Nevertheless, many of the lessons learned in our study (same interviewer, having participants choose the place and time of interview and identifying a proxy during the informed consent procedure) are most likely applicable to other sites when trying to recruit older newly-diagnosed cancer patients. The feedback obtained shows that older newly-diagnosed cancer patients are willing to participate in observational studies while receiving cancer treatment. There is a need to refine the recruitment and retention strategies for conducting observational research with older cancer patients.

## Key points for recruitment and retention

• Be present in the oncology clinics so that the physician is reminded to verify eligibility of the patient, to ask the patient permission to be contacted by a member of the research team. If the patient agrees to be contacted, the patient can briefly meet with you to know who will be calling or you can explain the study in person in the clinic.

• Provide all physicians involved in the patient recruitment with all the materials plasticized in bright colors to post in their exam rooms to remind them of the inclusion and exclusion criteria.

• Adjust the recruitment strategies for all the departments involved if necessary and evaluate continuously.

• During the informed consent procedure, ask consent to be able to contact a proxy to locate the patient for interviews as older cancer patients are often admitted in several health care institutions during the course of treatment.

• Be flexible when planning the interviews and offer home visits and transportation to the interviews.

• Organize interviews so that involved family members or friends can be present at the interviews.

• Adjust the materials/interviews to older persons with visual and hearing impairment.

• Use the same interviewer as much as possible.

• Send reminder letters and questionnaires in advance.

• Ask the patient consent to inform their treating physician of the results of the interview. Send a summary of the interview to all the physicians within a week of the interview which includes the list of important findings on the different measurement tools used including the established cut-offs, if available.

## Competing interests

The authors declare that they have no competing interests.

## Authors' contributions

MP designed the study, collected the data, performed the data analysis and interpretation and drafted the manuscript. JM participated in the design of the study, interpretation of the results of the data analyses, and helped to draft the manuscript. VG participated in the design of the study, in the data collection, in the interpretation of the results of the data analyses and helped to draft the manuscript. CW participated in the design of the study, data analysis and interpretation of the data, and helped to draft the manuscript. MM assisted in the data collection, was involved in the interpretation of the results of the data analyses and in the preparation of the manuscript. GB was involved in the study design and preparation of the manuscript. HB: was involved in the study design and preparation of the manuscript. All authors have read and approved the final version of the manuscript.

## Pre-publication history

The pre-publication history for this paper can be accessed here:

http://www.biomedcentral.com/1471-2407/9/277/prepub

## Supplementary Material

Additional file 1 **Overview of data collection and questionnaires used at each wave of data collection**. Overview of all the data collection cycles and what information was collected during each data collection cycle. Click here for file

Additional file 2 **Difference in feasibility of interviews by patients' characteristics**. Overview of the different aspects of feasibility of the interview by the characteristics of the patient. Click here for file
