# Supplementary material for: Participation of older newly-diagnosed cancer patients in an observational prospective pilot study: an example of recruitment and retention
Source: BMC Cancer. 2009 Aug 10;9:277. doi: 10.1186/1471-2407-9-277 (PMC3087334; doi:10.1186/1471-2407-9-277)
Supplement: Additional file 1 — Overview of data collection and questionnaires used at each wave of data collection. Overview of all the data collection cycles and what information was collected during each data collection cycle. [file 1471-2407-9-277-S1.doc]

**Overview of data collection and** questionnaires used at each wave of data collection

| **Domains (Questionnaire used)** | **Baseline interview before the start of cancer treatment** | **Telephone follow-up 6 and 18 weeks after the baseline interview** | **Face-to-face follow-up interview 3 and 6 months after the baseline interview** |
| --- | --- | --- | --- |
| **Sociodemographic information** |  |  |  |
| Age, education, country born, marital status, living conditions (with whom and at home/residential setting), social support available, income level | X |  |  |
| **Lifestyle factors** |  |  |  |
| Use of alcohol, smoking | X |  | X |
| **Health and Functional status** |  |  |  |
| Cognition (Mini Mental State Examination[52]and Montreal Cognition Assessment tool[53]) | X |  | X |
| Mood (Hospital Anxiety and Depression Scale[50]) | X |  | X |
| Strength (hand grip strength with a dynamometer 3 times[54]) | X |  | X |
| Mobility (4 meter gait speed test[55]) | X |  | X |
| Body weight and height was measured | X |  | X (only weight) |
| Weight loss in the previous 3 months was asked | X |  |  |
| Physical activity (3 items from the CHSA questionnaire [56]) | X |  | X |
| Instrumental activities of daily living (7 OARS items [46]) | X | X | X |
| Basic activities of daily living (6 Katz items[47]) | X | X | X |
| Functional limitations (7 Nagi items[48]) | X | X | X |
| Quality of life (EORTC QOL C30 questionnaire [45]) | X | X | X |
| Comorbid conditions (Functional Comorbidity Index[44]) | X |  | X |
| Use of medications (contact pharmacist) | X |  | X |
| **Domains (Questionnaire used)** | **Baseline interview before the start of cancer treatment** | **Telephone follow-up 6 and 18 weeks after the baseline interview** | **Face-to-face follow-up interview 3 and 6 months after the baseline interview** |
| Self-rated health[51] | X | X | X |
| **Ecog performance score** [49] | X |  | X |
| **Use of health care** |  |  |  |
| Use of personal care at home | X | X | X |
| Use of housekeeping services at home | X | X | X |
| Treatment received since previous interview |  | X | X |
| Visits to the general practitioner (how often and for what reason) |  | X | X |
| Visits to the emergency room (how often and for what reason) |  | X | X |
| Admissions to a hospital (how often and for what reason) |  | X | X |
| **Interviewer’s notes** | X | X | X |
| **Feedback regarding study** |  |  | X (only at 6 months) |
| **Obtained from medical chart** |  |  |  |
| Information with regard to diagnosis and stage | X |  |  |
| Treatment proposed | X |  |  |
| Treatment received |  |  | X |
| Treatment toxicity |  |  | X |
| Use of emergency room |  |  | X |
| Admission to hospital |  |  | X |
