# Supplementary material for: Participation of older newly-diagnosed cancer patients in an observational prospective pilot study: an example of recruitment and retention
Source: BMC Cancer. 2009 Aug 10;9:277. doi: 10.1186/1471-2407-9-277 (PMC3087334; doi:10.1186/1471-2407-9-277)
Supplement: Additional file 2 — Difference in feasibility of interviews by patients' characteristics. Overview of the different aspects of feasibility of the interview by the characteristics of the patient. [file 1471-2407-9-277-S2.doc]

**Difference in feasibility of interviews** by patients’ characteristics

| **Patient’s characteristic** | **Median Number of days in study** | **Median time* inter-view 1** | **Median time* inter-view 2** | **Median time* inter-view 3** | **Median time* inter-view 4** | **Median time* inter-view 5** | **% of post-poned inter-views** | **#Status interview 1**  **% completed in 1 meeting /in 2 meetings/ incomplete** | **Status interview 3**  **% completed in 1 meeting /in 2 meetings/ incomplete** | **Status interview 5**  **% completed in 1 meeting /in 2 meetings/ incomplete** |
| --- | --- | --- | --- | --- | --- | --- | --- | --- | --- | --- |
| **Age**  <74.1  >74.1 | 189.0  196.0 | 50  45 | 10  10 | 40  45 | 10  10 | 35  45 | 44.0  43.1 | 76.8/5.4/17.9  76.8/3.6/19.6 | 67.3/10.2/22.4  75.0/8.3/16.7 | 69.6/13/17.4  84.4/4.4/11.1 |
| **Sex**  Men  Women | 199  189 | 45  45 | 10  10 | 45  42.5 | 10  10 | 40  40 | 48.3  41.7 | 70.6/11.8/17.6  79.5/1.3/19.2 | 67.9/3.6/28.6  72.5/11.6/15.9 | 83.3/4.2/12.5  74.6/10.4/15 |
| **Living situation**  Lives alone  Does not live alone | 195.5  189.0 | 45  47.5 | 10  10 | 45  40 | 10  10 | 45  40 | 40.5  45.8 | 71.1/4.4/24.4  80.6/4.5/14.9 | 67.5/10.0/22.5  73.7/8.8/17.5 | 68.4/10.5/21  83.0/7.5/9.4 |
| **Born in Canada**  Yes  No | 190  189.5 | 45  50 | 10  10 | 45  40 | 10  12.5 | 40  40 | 36.8  52.3 | 78.5/4.6/16.9  81.5/9.3/9.3 | 81.5/9.3/9.3  58.1/9.3/32.6 | 82.4/11.8/5.9  70.0/5.0/25.0 |
| **Diagnosis**  Lung  Breast  Hematological malignancy  Colorectal | 188.0  188.0  195.0  207.5 | 55  45  40  45 | 10  10  12.5  10 | 45  40  45  50 | 10  10  15  10 | 42.5  40  47.5  47.5 | 56.5  39.5  45.0  33.3 | 77.8/7.4/14.8  95.5/0/4.5  66.7/4.8/28.6  45.0/10.0/45.0 | 62.5/8.7/26.1  82.9/9.8/7.3  61.1/5.6/33.3  60.0/13.3/26.7 | 87.7/0/13.3  100/0/0  90.9/0/9.1  100/0/0 |
| **Extent of disease**  Early  Advanced | 189  196 | 45  50 | 10  10 | 42.5  45 | 10  10 | 40  40 | 35.6  54.8 | 79.4/1.6/19.0  73.5/8.2/18.4 | 80.4/7.3/12.7  59.5/11.9/28.6 | 87.3/5.5/7.3  61.1/13.9/25 |
| **Ecog**  0  1  >2 | 188  198  200 | 45  50  50 | 10  10  10 | 45  40  50 | 10  10  10 | 40  45  52.5 | 35.3  57.9  64.3 | 81.9/5.6/12.5  77.3/0/22.7  55.6/5.6/38.9 | 72.7/10.6/16.7  66.7/11.1/22.2  69.2/0/30.8 | 75.4/12.3/12.3  86.7/0/13.3  72.7/0/27.3 |
| **Mood impaired**  No  Yes | 189.5  200.5 | 45  50 | 10  15 | 42.5  40 | 10  12.5 | 40  40 | 38.5  58.3 | 81.5/2.5/16.0  76.9/7.7/15.4 | 70.4/12.7/16.9  77.3/0/22.7 | 75.4/10.1/14.4  78.9/5.3/15.8 |
| **Patient’s characteristic** | **Median Number of days in study** | **Median time* inter-view 1** | **Median time* inter-view 2** | **Median time* inter-view 3** | **Median time* inter-view 4** | **Median time* inter-view 5** | **% of post-poned inter-views** | **#Status interview 1**  **% completed in 1 meeting /in 2 meetings/ incomplete** | **Status interview 3**  **% completed in 1 meeting /in 2 meetings/ incomplete** | **Status interview 5**  **% completed in 1 meeting /in 2 meetings/ incomplete** |
| **Cognitive impairment**  No  Yes | 189  190.5 | 45  50 | 10  10 | 45  42.5 | 10  10 | 40  40 | 39.4  56.0 | 77.5/5.0/17.5  92.3/0/7.7 | 71.4/10.0/18.6  81.8/9.1/9.1 | 74.6/10.4/14.9  90.0/5.0/5.0 |
| **IADL disability**  No  Yes | 188  198.5 | 45  50 | 10  12.5 | 42.5  45 | 10  10 | 40  50 | 34.8  62.5 | 82.2/5.5/12.3  66.7/2.6/30.8 | 71.2/10.6/18.2  71.0/6.5/22.6 | 77.8/11.1/12.1  75.0/3.6/21.5 |
| **ADL disability**  Yes  No | 189.5  198 | 45  47.5 | 10  15 | 40  57.5 | 10  15 | 40  55 | 46.7  18.2 | 78.0/5.0/17.0  66.7/0/33.3 | 71.3/8.0/20.7  70.6/20.0/10.0 | 76.2/8.3/15.0  85.7/14.3/0 |
| **Number of comorbid condition**  0  1  2  3  4+ | 195.5  182.5  189.5  194.0  201.5 | 40  45  50  40  52.5 | 10.5  10  10  10  15 | 45  40  45  42.5  50 | 10  10  10  10  10 | 47.5  40  40  40  45 | 54.5  28.0  40.9  30.8  63.2 | 60.9/17.4/21.7  81.5/0/18.5  85.2/0/14.8  84.6/0/15.4  72.7/4.5/22.7 | 63.6/9.1/27.3  83.3/4.2/12.5  75.0/10.0/15.0  76.9/15.4/7.7  55.6/11.1/33.3 | 70.0/15.0/15.0  83.3/8.3/8.3  80.0/5.0/15.0  84.6/7.7/7.7  64.3/7.1/28.6 |

* Time in minutes

# for interview 2 and 4 (telephone follow-up), almost all the interviews were complete (for the few proxy interviews not all questionnaires are possible) and conducted in 1 and therefore not added in the table as there was no difference based on patients’ characteristics.
